# Circulating MicroRNAs in Maternal Blood as Potential Biomarkers for Fetal Hypoxia In-Utero

**DOI:** 10.1371/journal.pone.0078487

**Published:** 2013-11-25

**Authors:** Clare L. Whitehead, Wan Tinn Teh, Susan P. Walker, Cheryl Leung, Luke Larmour, Stephen Tong

**Affiliations:** 1 Translational Obstetrics Group, Department of Obstetrics and Gynaecology, Mercy Hospital for Women, Heidelberg, Victoria, Australia; 2 Department of Obstetrics and Gynaecology, University of Melbourne, Royal Hospital for Women, Carlton, Victoria, Australia; 3 Department of Obstetrics and Gynaecology, University of Melbourne, Mercy Hospital for Women, Heidelberg, Victoria, Australia; 4 Department of Obstetrics and Gynaecology, Monash Medical Centre, Clayton, Victoria, Australia; VU University Medical Center, The Netherlands

## Abstract

Stillbirth affects 1 in 200 pregnancies and commonly arises due to a lack of oxygen supply to the fetus. Current tests to detect fetal hypoxia in-utero lack the sensitivity to identify many babies at risk. Emerging evidence suggests that microRNAs derived from the placenta circulate in the maternal blood during pregnancy and may serve as non-invasive biomarkers for pregnancy complications. In this study, we examined the expression of miRs known to be regulated by hypoxia in two clinical settings of significant fetal hypoxia: 1) labour and 2) fetal growth restriction. Six miRs (miR 210, miR 21, miR 424, miR 199a, miR 20b, and miR 373) were differentially expressed in pregnancies complicated by fetal hypoxia. In healthy term pregnancies there was a 4.2 fold increase in miR 210 (p<0.01), 2.7 fold increase in miR 424 (p<0.05), 2.6 fold increase in miR 199a (p<0.01) and 2.3 fold increase in miR 20b (p<0.05) from prior to labour to delivery of the fetus. Furthermore, the combined expression of miR 21 and miR 20b correlated with the degree of fetal hypoxia at birth determined by umbilical cord lactate delivery (r = 0.79, p = 0.03). In pregnancies complicated by severe preterm fetal growth restriction there was upregulation of the hypoxia-regulated miRs compared to gestation-matched controls: 3.6 fold in miR 210 (p<0.01), 3.6 fold in miR 424 (p<0.05), 5.9 fold in miR 21 (p<0.01), 3.8 fold in miR 199a (p<0.01) and 3.7 fold in miR 20b (p<0.01). Interestingly, the expression of miR 373 in gestation matched controls was very low, but was very highly expressed in FGR (p<0.0001). Furthermore, the expression increased in keeping with the degree of in-utero hypoxia estimated by fetal Doppler velocimetry. We conclude quantifying hypoxia-regulated miRs in the maternal blood may identify pregnancies at risk of fetal hypoxia, enabling early intervention to improve perinatal outcomes.

## Introduction

Fetal hypoxia ultimately leads to fetal death and disability. Hypoxia may occur acutely during labour and birth, or develop gradually across pregnancy in cases of fetal growth restriction (FGR) due to placental dysfunction.

Fetal growth restriction accounts for 50% of unexplained stillbirths and is strongly associated with permanent neurological disability in survivors [1], [2].There are no effective intrauterine therapeutic options for FGR, and management relies on early detection and timely delivery before stillbirth occurs [3], [4]. Unfortunately, many cases of stillbirth arise due to a failure to detect the presence of FGR, and the degree of hypoxia present in utero.

Fetal hypoxia may also occur acutely during labour in an otherwise healthy baby, as a result of cord occlusion or restricted placental perfusion. 1 in 200 newborns are affected by perinatal asphyxia while 2 per 1000 newborns develop hypoxic-ischaemic encephalopathy (HIE), which accounts for 1 million neonatal deaths globally per year [5]. Of those who survive the initial perinatal insult, at least 25% will develop long-term neurodevelopmental sequelae [6].

Therefore, clinicians utilize a combination of tests to assess fetal well-being to determine whether the fetus is at risk of succumbing to hypoxia. These include: clinical judgment; ultrasound assessment of fetal growth, fetal movement and fetal vessel velocimetry; and recording fetal heart rate patterns [7]. However, this current strategy fails to recognize as many as 85% of growth restricted fetuses and although intrapartum monitoring has reduced the incidence of neonatal seizures, the rates of cerebral palsy and neonatal death have not decreased since its introduction. Therefore the development of an alternative assessment of fetal hypoxia is greatly needed (5).

The exciting discovery that microRNAs circulate in the blood and may reflect gene expression in a distant tumour, has led to enthusiasm for the role of circulating miRs as biomarkers for cancer detection and prognosis [8]. More recently, circulating placental specific miRs have been identified in the maternal blood during pregnancy, which may reflect both physiological and pathological placental conditions [9]. This offers a potential new avenue to develop a screening test for pregnancy complications. miRNAs are 19–25 nucleotide regulatory non-coding RNAs that play important roles in the regulation of a wide variety of genes by binding to the 3′ untranslated regions of the target mRNA [8]. Hypoxia has a profound effect on gene regulation and miRNAs play a vital role in the cellular response to hypoxia [10]. Therefore, we reasoned that measuring hypoxia regulated circulating miRNAs in the maternal blood may be a promising strategy to identify biomarkers for fetal hypoxia in-utero. In this study we examine the expression of a panel of hypoxia induced miRs in the maternal blood when the fetus was exposed to: 1) acute hypoxia during labour and 2) chronic hypoxia associated with fetal growth restriction.

## Materials and Methods

### Study Subjects

All subjects were recruited from two tertiary hospitals in Melbourne, Australia (Mercy Hospital for Women and Monash Medical Centre) between 2008–2012. Written informed consent was obtained prior to inclusion and the study protocol was approved by both institutions’ Human Research and Ethics Committees. Two separate cohorts were recruited: acute hypoxia in labour and chronic hypoxia in FGR.

### Acute Hypoxia during Labour

Maternal whole blood samples were prospectively collected from consecutive women undergoing induction of labour at term (n = 8). A second intravenous cannula was inserted for serial blood collection for the study: prior to the onset of uterine contractions and at the moment of delivery (defined as the time when the fetal head is crowning at the vaginal opening and birth was imminent). Fetal hypoxic status was determined by measuring umbilical artery blood lactate levels collected at delivery.

### Chronic Hypoxia in FGR

Maternal whole blood samples were collected from women carrying a preterm severely growth restricted fetus (n = 12) and gestation matched controls (n = 12). Severe preterm FGR was defined as customized birthweight <10^th^ centile (www.gestation.net, Australian dataset) requiring iatrogenic delivery before 34 weeks with antenatal evidence of uteroplacental insufficiency (asymmetrical growth+abnormal umbilical artery Doppler velocimetry, +/− oligohydramnios, +/− abnormal fetal vessel velocimetry). FGR due to infection, chromosomal or congenital abnormalities, and multiple pregnancies were excluded. Preterm control blood samples were collected from women with an appropriately grown fetus, at a gestation matched to the FGR cases, but who subsequently delivered appropriately grown (birthweight 20–80^th^ centile) neonates at term without obstetric complications.

### Sample Collection and miRNA Preparation

Peripheral whole blood samples (2.5 ml) were collected in PAXgene blood RNA tubes (PreAnalytix, Hombrechtikon, Switzerland). As per manufacturers instructions, they were stored at room temperature for 24 h, transferred to −20°C for 24 h, and then stored at −80°C until processing.

miRNA was extracted from peripheral whole maternal blood using the PAXgene Blood miRNA kit (PreAnalytix) according to manufacturer’s instructions, as previously described [11]. Genomic DNA was removed using DNAse treatment, and total RNA eluted and stored at −80°C if not used immediately. The concentration of total RNA was quantified by NanoDrop 1000 (Nanodrop).

### Real Time Quantitative RT-PCR

miRNA Taqman assays (Applied Biosystems) were used for the quantification of the miRNAs according to manufacturer’s instructions. 50 ng of total RNA was reverse transcribed for each target miRNA with the pool of housekeeping miRNAs: RNU-48 and RNU-6b. qPCR was performed in triplicate, with multiple negative controls, using TaqMan Universal PCR Master Mix on a CFX 384 (BioRad, Foster CA) with the following cycling conditions: 50C for 2 mins, 95C for 10 mins, and 40 cycles of 95C for 15 sec, 60C for 1 min. Fold changes in expression were determined by the comparative CT method normalized against the mean expression of RNU-48 and RNU-6b as reference genes.

### Statistical Analysis

All data was statistically analyzed using R version 2.12.0 (http://www.r-project.org) or Graphpad Prism v 5(GraphPad Software Inc., San Diego, CA). Differences in RT-PCR gene expression was assessed using Mann–Whitney U or Kruskal –Wallis test, or Wilcoxon signed rank test as appropriate. Patient characteristics were compared using χ^2^ where appropriate. Data was presented as mean +/− SD. Significance was defined as p<0.05.

## Results

### Expression of Hypoxia-induced miRNAs due to Acute Fetal Hypoxia in Labour

During labour there is a cessation of maternal blood flow to the myometrium with each uterine contraction. As a result, there is progressive hypoxia of the placenta and fetus as labour advances, rendering some fetuses acidotic at birth. Labour is therefore effectively an *in vivo* model of acute human fetal hypoxia.

**Figure 1:**
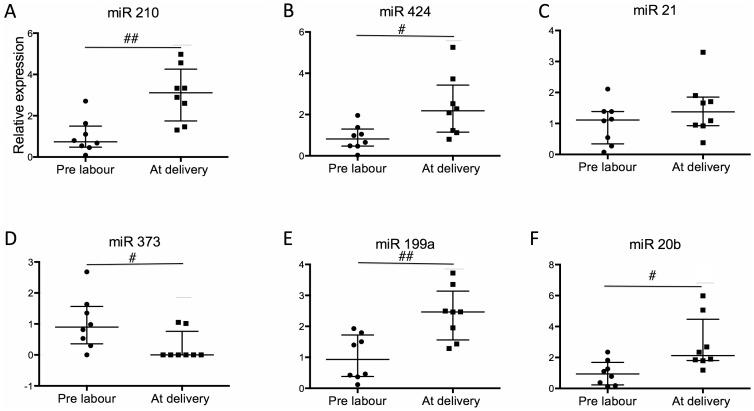
Expression of hypoxia induced miRNA in maternal blood from women in labour. (n = 8). miRNA expression of (A) miR 210, (B) miR 424, (C) miR 21, (D) miR 373, (E) miR 199a, (F) miR 20b. Data is presented as median +/− IQR. Wilcoxon matched-pairs rank test was used for statistical analysis. #p<0.05, ##p<0.01. Normalised against RNU 48 and RNU 6b.

We first examined whether hypoxia – induced miRNAs were increased in maternal blood across the duration of labour. The patient characteristics are summarized in Table 1. We focused on the expression of six miRNAs known to be induced by hypoxia: miR 210, miR 424, miR 21, miR 373, miR 199a and miR 20b. Compared to before induction of labour and the onset of uterine contractions, at delivery we found a 4.2 fold increase in miR 210 (prelabour median 0.74 vs delivery median 3.11, p<0.01), a 2.7 fold increase in miR 424 (0.82 vs 2.18, p<0.05), a 2.6 fold increase in miR 199a (0.93 vs 2.47, p<0.01) and a 2.3 fold increase in miR 20b (0.94 vs 2.12, p<0.05). In contrast, there was a reduction in the expression of miR 373 across labour (prelabour median 0.89 vs delivery median 0.00, p<0.05). Labour did not lead to a significant difference in the expression of miR 21. (Fig. 1) Thus, we conclude that a number of hypoxic-induced miRNAs increase across labour.

**Table 1:** Clinical characteristics for acute hypoxia cohort: in labour.

		High lactate (n = 4)	Low lactate (n = 4)
Age (yrs)		27.3 (3)	28.2 (5)
Parity (% Primiparous)		100	100
Gestational Age (weeks)		40+1 (4)	40+2 (7)
Delivery	Vaginal	50	75
Mode (%)	Instrumental	50	25
	Caesarean	0	0
Birthweight (gms)		3154 (570)	3408 (480)
1 minute apgar		9 (8–9)	9 (7–9)
5 minute apgar		9 (9–9)	9 (9–9)
Cord lactate (mmol/L)		6.4 (1)	3.4 (1)
Admission to neonatal unit (%)		25	0

Data is presented as mean (SEM), median (IQR) or percentage.

**Figure 2:**
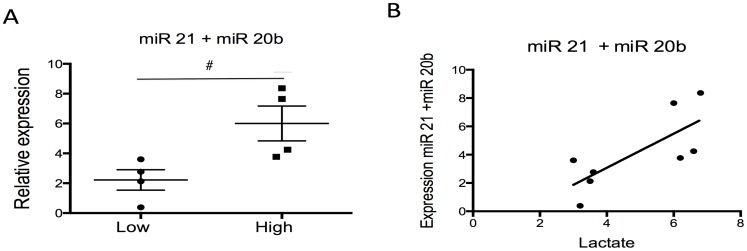
Combined expression of miR 21 and miR 20b in maternal blood sampled the moment before delivery. (**A**) Combined expression comparing those women delivering a fetus with a high lactate (High >6 mmol/L) to those delivering a fetus with a low lactate (Low <4 mmol/L). Data is presented as median +/− IQR. Mann – U Whitney test was used for statistical analysis. #p<0.05, ##p<0.01. Normalised against RNU 48 and RNU 6b (**B**) Correlation of combined expression of miR 21 and miR 20b to umbilical cord blood lactate level. Spearman’s rank correlation coefficient (r = 0.56, p = 0.03).

The presence of significant hypoxia in tissues can result in elevated blood levels of lactate. Lactate is produced when tissues deprived of oxygen switch from oxidative phosphorylation to anaerobic metabolism. Thus, elevated lactate in umbilical cord blood reflects significant fetal hypoxia, and indeed, higher levels have been shown epidemiologically to be associated with neonatal encephalopathy. We chose a cord lactate >6 mmol/L to represent a hypoxic fetus in our cohort, as this has been suggested as the cut-off to predict which fetuses will develop moderate-severe HIE [12]. In our study, no single miR was able to discriminate between those fetuses with a low lactate (<4 mmol/L) or high lactate (>6 mmol/L). However, there was a 3 fold increase in the combined expression of miR 21 and miR 20b in those fetuses with a high lactate (median expression low lactate 2.45 vs high lactate 5.94, p<0.05). When the expression of miR21 and miR 20b were combined, there was a significant correlation (Spearman’s rank) with umbilical cord lactate levels at delivery (r = 0.79, p = 0.03). (Fig. 2).

### Expression of Hypoxia-induced miRNAs due to Chronic Hypoxia in FGR

Impaired placental perfusion due to placental insufficiency leads to placental hypoxia and a severely growth restricted fetus. We next examined the expression of hypoxia-induced miRNAs in maternal blood from women with severe FGR compared to healthy gestation matched controls. The patient characteristics are summarized in Table 2.

**Table 2:** Clinical characteristics for chronic hypoxia cohort: severe preterm FGR.

	FGR (n = 12)	Gestation matched controls (n = 12)
Maternal age (yrs)	31.4 (4.7)	30.3 (2.5)
Parity (% primiparous)	67	75
Gestational age at delivery (weeks)	30+1 (3)	40+2 (1.4)
Gestational age at sampling (weeks)	30+1 (3)	30+2 (2.0)
Birthweight (gms)	876 (285)	3670 (64)
Customised birth centile (%)	3 (3)	52 (7)
Perinatal death (%)	17	0
Pre-eclampsia (%)	33	0
Umbilical artery Doppler velocimetry waveform (%):		
REDF	17	0
AEDF	25	0
↑PI	58	0
Normal	0	100

Data is presented as mean (SEM), median (IQR) or percentage.

REDF: reversed end diastolic flow. AEDF: absence end diastolic flow. ↑PI: increased pulsitility index with positive end diastolic flow. (The progression from increased PI to REDF in the umbilical artery represents increasing placental resistance and therefore is an indirect measure of the severity of fetal growth restriction due to placental insufficiency).

**Figure 3:**
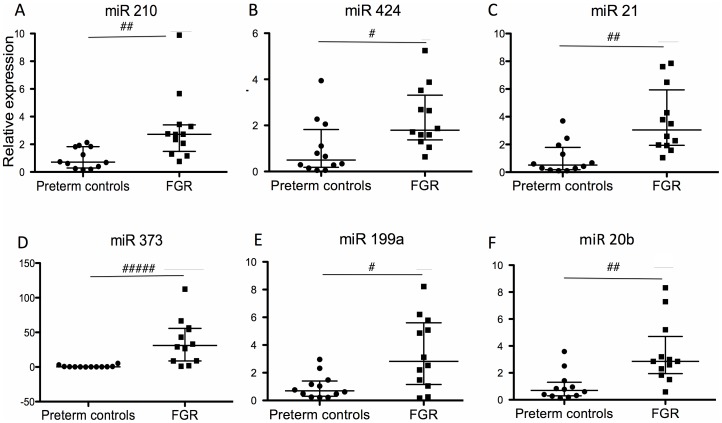
Expression of hypoxia induced miRNA in maternal blood from women with severe preterm FGR (n = 12) compared to gestation matched controls (N = 12). miRNA expression of (A) miR 210, (B) miR 424, (C) miR 21, (D) miR 373, (E) miR 199a, (F) miR 20b. Data is presented as median +/− IQR. Mann – U Whitney test was used for statistical analysis. #p<0.05, ##p<0.01, ###p<0.001. Normalised against RNU 48 and RNU 6b.

Of interest, the expression of miR 373 in gestation matched controls was very low, but was very highly expressed in FGR, with a 29 fold increase (control median 31.1 vs FGR median 0.93, p<0.0001). Interestingly, this significant increase observed in FGR, a situation of chronic hypoxia, was opposite to the trends seen in our labour ward study, where there was a reduction in expression with acute hypoxia. There was also a 3.6 fold increase in miR 210 (control median 0.71 vs FGR median 2.60, p<0.01), 3.6 fold increase in expression of miR 424 (0.50 vs 1.79, p<0.05), 5.9 fold increase in miR 21 (0.51 vs 3.04, p<0.01), 3.8 fold increase in miR 199a (p<0.01) and 3.7 fold increase in miR 20b (0.79 vs 2.81, p<0.01) (Fig. 3).

Ultrasound velocimetry of the umbilical artery is used clinically to determine the severity of preterm FGR and the likelihood of fetal hypoxia. Doppler assessment of the umbilical artery evaluates blood flow from the fetus to the placenta and therefore reflects placental vessel resistance and end organ function of the fetus [13]. Early in placental dysfunction, there is increased placental resistance which can be measured in the umbilical artery using the pulsatility index (UA PI). As the degree and duration of fetal hypoxia progresses, the fetus attempts to compensate by redistributing its peripheral circulation. As a result, there is progressive loss of forward flow in diastole in the umbilical artery. This is described as Absent or Reversed End Diastolic Flow (AREDF), which has a strong association with fetal hypoxemia and perinatal mortality. This progression in severity of Doppler indices in the umbilical artery, allow clinicians to estimate the severity of disease and time delivery accordingly [3], [14].

**Figure 4:**
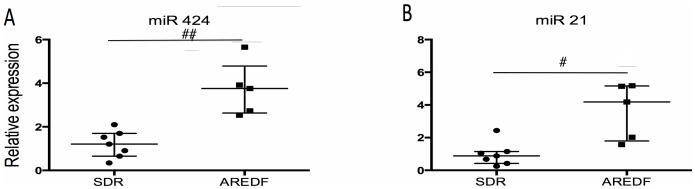
Expression of hypoxia induced miRNA in maternal blood from women with severe preterm FGR with increased pulsatility index (PI) compared to AREDF, representing the more severely hypoxic cohort. miRNA expression of (A) miR 424 and (B) miR 21. Data is presented as median +/− IQR. Mann – U Whitney test was used for statistical analysis. #p<0.05, ##p<0.01, ###p<0.001. Normalised against RNU 48 and RNU 6b.

We therefore divided the FGR cohort into those fetuses who had a raised pulsatility index (associated with less severe hypoxia) and compared miRNA expression to those with AREDF (associated with more severe hypoxia). There was a trend to increased expression with all hypoxia induced miRs with AREDF but a significant increase was only observed in miR 424 (1.21 vs 3.76, p<0.01) and miR 21 (0.89 vs 4.18, p<0.05) in those FGR with AREDF (Fig. 4).

## Discussion

Placental transfer of oxygen to the fetus is essential for growth and survival. Birth asphyxia and placental insufficiency lead to placental and fetal hypoxia, and are the commonest causes of stillbirth, neonatal morbidity and long-term neurological sequelae [2], [6], [15]. The only effective treatment is to deliver the fetus before significant hypoxia occurs [7]. However, the current clinical tools available to clinicians are unable to accurately diagnose fetal hypoxia or quantify its severity [16].

In this study, we have identified a novel approach to non-invasively diagnose fetal hypoxia by measuring circulating miRNAs in the maternal blood. We demonstrate that a panel of hypoxia-induced miRNAs are detectable in the maternal blood, and that their expression changes when the fetus is subjected to both acute and chronic hypoxia. Furthermore, quantification of hypoxia-induced miRNAs correlated with the degree of in-utero hypoxia.

Electronic monitoring of fetal heart rate patterns is currently the mainstay of assessment of fetal well-being during labour, but has a low predictive value for fetal hypoxia. As a result, research into adjunctive methods of assessing the degree of fetal hypoxia has been recommended [17]. A non-invasive measure of fetal hypoxia would be ideal, yet we are not aware of any known maternal biomarker of fetal hypoxia. Strikingly, we demonstrated that the combination of miR 21 and miR 20b correlated with the degree of fetal hypoxia at birth. This proof of concept study is the first study to profile dynamic changes in miRNA expression. However, numbers were low in this study and a larger prospective study is required to validate this finding. However, current miRNA assay processing time prohibits translation of this potential test as a biomarker of acute hypoxia in labour.

In addition, we have demonstrated that hypoxia-induced miRNAs differentiated severe FGR pregnancies from healthy gestation matched controls. This is in contrast to current antenatal surveillance, which fails to detect 75–85% of FGR cases, pregnancies at considerable risk of antepartum stillbirth [18]. Once FGR is detected, ultrasound surveillance improves perinatal outcomes, with delivery of the fetus recommended when fetal hypoxia is suspected [19]. We found that the quantification of the hypoxia-induced miRNAs correlated with the degree of fetal hypoxia associated with abnormal fetal velocimetry. It is possible that measurement of miRNAs in maternal blood may therefore form the basis of a future test to determine the degree of fetal hypoxia in FGR.

The discovery of miRNAs in the maternal circulation has yielded much potential for biomarker discovery [9]. miRNA in the maternal circulation is largely derived from the placenta, present from early pregnancy, increases across gestation and disappears after delivery [20]. There is a strong correlation between placental miRNA distribution and both maternal and fetal circulating miRNAs expression, but no correlation with miRNAs in the paternal or non-pregnant circulation [21]. The exact mechanism by which miRNAs enter the maternal circulation is largely unknown but they may be released from the syncytiotrophoblast by exosomes [22]. These circulating miRNAs offer advantages as biomarkers over circulating RNA and proteins as they are largely intact, protected from degradation and more stable for detection [9].

Several studies have demonstrated altered expression of miRNAs in placental tissue but although these studies provide biological insight, their clinical utility is limited by the necessity of awaiting delivery to obtain placental tissue [23], [24], [25], [26]. Circulating miRNAs overcome this disadvantage, enabling profiling of physiological and pathological changes in the placenta, whilst the fetus remains in-utero. However, few studies are yet to look at circulating miRNA in placental disorders, specifically pre-eclampsia and FGR [27], [28], [29].

In this study we choose to screen hypoxia–induced miRNAs as potential biomarkers of fetal hypoxia. The cellular response to hypoxia results in induction of a complex set of gene regulatory mechanisms, largely controlled by the hypoxia inducible factors (HIFs). More than 90 microRNAs have been identified that are induced by hypoxia and modulate the cellular response to hypoxia via HIFs [10], [30]. Of these, miR 210 is the best described as it is ubiquitously induced in all cell types in response to hypoxia, and directly responsible for the adaptation of multiple cellular processes [10]. Furthermore, miR 210 has been consistently shown to be upregulated in placenta from pre-eclamptic pregnancies, although its expression in FGR has yet to be described [23], [25], [26], [31]. In addition to miR 210, a handful of miRNAs induced by hypoxia have been identified that are regulated in a HIF-dependent manner. In this study we selected 5 HIF-dependent miRNAs whose expression in placenta is largely unknown to screen for potential biomarkers in fetal hypoxia. Table 3 describes the function and association with pregnancy complications of these hypoxia induced miRNAs in this study.

**Table 3:** Hypoxia induced microRNA and their expression in pregnancy complications.

microRNA	Hypoxia regulation via HIF1α	Expression in pre-eclampsia	Expression in FGR
**miR 210**	Upregulated by HIF1α [10]	↑ placenta [24], [25], [26], [31], [32] ↑ plasma [33]	↑ placenta [34]
**miR 21**	Upregulated by HIF1α [35]	unknown	↓ placenta [36]
**miR 424**	Increases HIF1α [37]	unknown	In plasma [29]
**miR 373**	Upregulated by HIF1α [38]	unknown	unknown
**miR 20b**	Upregulated by HIF1α and increases HIF1α [39]	↑ placenta [40]	unknown
**miR 199a**	Binds to and reduces HIF1α [35]	unknown	unknown

Although our results are promising several limitations are recognized. The small sample size requires further validation in a larger cohort to determine the diagnostic potential of such an approach. The speed at which analysis can be performed currently prohibits the potential of such a test as a biomarker for acute hypoxia but may still be useful for the detection of chronic hypoxia in cases of severe FGR.

In conclusion, we have demonstrated an elevation in hypoxia-induced miRNAs in both acute and chronic fetal hypoxia that may be a promising approach to clinically assess fetal hypoxic status in-utero. This may help clinicians to identify hypoxic fetuses allowing timely intervention to improve perinatal outcomes and decrease the rates stillbirth.
